# U.S. synthetic residential building telemetry dataset generated with EnergyPlus

**DOI:** 10.1016/j.dib.2026.113327

**Published:** 2026-10-08

**Authors:** You-Jeong Kim, Yun Kyu Yi

**Affiliations:** School of Architecture, University of Illinois at Urbana-Champaign, 117 Temple Hoyne Buell Hall, 608 Lorado Taft Dr, Champaign, IL, 61820, USA

**Keywords:** Synthetic building telemetry, U.S. Residential building stock, Smart thermostat data, HVAC operation, EnergyPlus

## Abstract

High-resolution residential telemetry, including smart thermostat data, is increasingly used to develop data-driven models for building energy analysis and HVAC performance assessment. However, field telemetry often lacks detailed information on building characteristics and known physical parameters, limiting rigorous model development and validation. This article presents a U.S. synthetic residential building telemetry dataset generated using EnergyPlus that combines high-resolution time-series telemetry with fully known building, envelope, infiltration, and HVAC parameters. It comprises 540 unique models of single-family detached and attached homes across the 48 contiguous states and 13 ASHRAE climate zones. The models were generated using probability distributions of building and HVAC characteristics derived from the ecobee Donate Your Data (DYD) program and the U.S. residential building stock represented by ResStock. Statistical case weights are provided for the 540 unique models, defining a probability-weighted analytical population of 1200 residential cases. For each model, the dataset provides an EnergyPlus input file and a full year of synthetic telemetry at 5-minute intervals in Parquet format. The telemetry includes indoor and outdoor environmental conditions, thermostat setpoints, HVAC operation and runtime, infiltration and ventilation, heating and cooling rates, and energy-consumption variables. The dataset also includes building characteristics, simulation metadata, and weather-station location. Comparison of HVAC-runtime patterns with ecobee field telemetry indicates that the synthetic data reproduce broad climate-dependent patterns in residential HVAC operation while retaining known model inputs and physical characteristics. The dataset can support the development and benchmarking of surrogate and telemetry-based models, evaluation of retrofit and HVAC-sizing scenarios, and analysis of residential energy performance at national, state, and climate-zone scales.

Specifications Table

**Table:** 

Subject	Engineering & Materials science
Specific subject area	Synthetic residential building-energy models and high-resolution heating, air conditioning, and ventilation (HVAC) system telemetry
Type of data	Table (.csv), EnergyPlus input data file (.idf); compressed Parquet telemetry (.gz.parquet)
Data collection	Building and HVAC attributes were derived from probability distributions informed by U.S. smart-thermostat metadata (ecobee Donate Your Data) and residential building-stock data (ResStock). A probability-informed case-generation and ranking procedure retained 540 unique configurations. Each model was simulated in EnergyPlus version 9.5 for one year using TMY, TMY2, or TMY3 weather data. Five-minute telemetry outputs were processed and stored in compressed Parquet format. Case probabilities were normalized and scaled to represent 1000 detached and 200 attached homes.
Data source location	Institution where the synthetic data were generated: University of Illinois Urbana-Champaign, Champaign, Illinois, USA. Primary sources: ecobee Donate Your Data metadata; National Renewable Energy Laboratory’s ResStock Housing Characteristics Probability Distributions; EnergyPlus weather data; and, for the non-heat-pump heating-system fuel assumption only, the U.S. Energy Information Administration Residential Energy Consumption Survey.
Data accessibility	Repository name: Zenodo Data identification number: https://doi.org/10.5281/zenodo.23005231 Direct URL to data: https://zenodo.org/records/23005231
Related research article	None.

## 1 Value of the Data

• The dataset provides full-year residential telemetry at five-minute resolution with known building-envelope, infiltration, HVAC-system, and operational inputs, offering a controlled complement to field smart-thermostat data in which many physical characteristics are unobserved.
• The 540 unique EnergyPlus models cover detached and attached homes across the 48 contiguous U.S. states and 13 ASHRAE climate zones; statistical case weights define a probability-weighted analytical population of 1200 residential cases.
• Because the models were generated from probability distributions informed by ecobee Donate Your Data (DYD) and National Renewable Energy Laboratory (NREL) ResStock, the dataset supports national-scale benchmarking while remaining computationally manageable.
• Researchers can use the models and telemetry to evaluate the scalability and generalizability of surrogate building-energy models, benchmark modeling methods against known physical parameters, test HVAC control strategies, assess retrofit and equipment-sizing scenarios, and analyze energy performance and thermal comfort across locations and building types.

## 2 Background

High-resolution smart-thermostat telemetry has become an important resource for building-energy research because it provides large-scale observations of indoor conditions, outdoor weather, thermostat setpoints, and heating, air-conditioning, and ventilation (HVAC) system operation. The ecobee Donate Your Data (DYD) program [1] has supported predictive modeling, thermal characterization, HVAC performance assessment, and control research. However, the thermophysical characteristics of monitored homes, such as envelope thermal properties, infiltration, equipment efficiencies, and detailed system configurations, are generally unknown. This limits rigorous evaluation of whether telemetry-based models recover physically meaningful parameters and restricts controlled testing of algorithms against known ground truth.

National building-stock resources provide a complementary source of information. National Renewable Energy Laboratory (NREL) ResStock characterizes detailed distributions of U.S. residential building attributes [2], but the full stock contains approximately 550,000 models and is computationally intensive for repeated model development and sensitivity analysis. This motivated the development of a reduced, probability-informed synthetic residential stock that combines smart-thermostat metadata with national building-stock characteristics and provides high-resolution telemetry with known physical and system properties.

This data article describes the development of a reduced synthetic residential building stock, the associated EnergyPlus models and five-minute telemetry outputs, and the methods used for case generation, simulation, and dataset evaluation.

## 3 Data Description

The released dataset contains three core case-level components: case metadata (case_description.csv), EnergyPlus input files (*.idf), and synthetic telemetry (*.gz.parquet), together with supporting documentation, weather mapping, and QA/QC files, as described in Table 1. A common case identifier (from “001″ to “540”) connects the case-description record, the EnergyPlus model, and the corresponding telemetry file. The complete dataset described in this article is publicly available through Zenodo [3].

**Table 1:** Overview of the dataset components, file formats, and contents.

Component	Format	Contents
Case description	case_description.csv	Building and simulation metadata for the 540 unique EnergyPlus models, including building, envelope, infiltration, and HVAC information, including source properties and corresponding EnergyPlus inputs.
EnergyPlus input	*.idf	Complete EnergyPlus version 9.5 model definitions and simulation assumptions for each of the 540 unique cases.
Synthetic telemetry	*.gz.parquet	One full year of EnergyPlus outputs at five-minute intervals for each case (105,120 records per complete case).
Dictionary	dictionary.csv	Variable names, descriptions, data source, and units for the fields reported in case_description.csv.
Weather data summary	weather_data_summary.csv	Mapping of the selected ecobee city, selected EnergyPlus weather station, ASHRAE climate zone, and exact weather file used for each state.
Miscellaneous data	*.csv	Supporting QA/QC and validation files: HVAC COP updates (idfs_v2_cop_change_report.csv), telemetry quality checks (telemetry_data_quality_summary.csv, telemetry_data_quality_variables.csv), and CPM climate-zone summaries (cpm_climate_zone_summary.csv).

The case_description.csv file defines the residential building cases used to generate the synthetic dataset. It contains 540 unique EnergyPlus model configurations, with each row representing one model identified by case_ID. The cases cover the 48 contiguous U.S. states, with one representative weather station assigned to each state. The file includes 360 single-family detached and 180 single-family attached models. It reports building, envelope, infiltration, HVAC, and case-weight information, including both source properties and corresponding EnergyPlus inputs. Table 2 summarizes the variables included in case_description.csv, while detailed definitions, units, data sources, applicability, and transformation or mapping information are provided in dictionary.csv.

**Table 2:** Variables and definitions in case_description.csv.

Variable	Description	Unit
case_ID	Unique identifier for each synthetic residential building configuration	-
house_type	Residential building type: single-family detached or attached	-
state	U.S. state represented by the case	-
weather_station_location	Weather station used to assign local weather conditions	-
structure_type	Primary construction type	-
horizontal_location	Position of an attached unit: middle or side; not applicable to detached homes	-
vintage	Building construction-period category	-
wall_source_R_value	Source wall-insulation thermal resistance from ResStock	ft²·°F·h/BTU
wall_insulation_thickness	Wall insulation thickness used in the EnergyPlus model	m
ceiling_source_R_value	Source ceiling/roof-insulation thermal resistance from ResStock	ft²·°F·h/BTU
ceiling_insulation_thickness	Ceiling/roof insulation thickness used in the EnergyPlus model	m
window_glazing	Window/glazing category from ResStock	-
window_U_factor	Window thermal transmittance used in the EnergyPlus model	W/m²·K
window_SHGC	Window solar heat gain coefficient used in the EnergyPlus model	-
infiltration_source_ACH50	Source airtightness at 50 Pa from ResStock	1/h at 50 Pa
infiltration_rate	Natural infiltration air-change rate used as the EnergyPlus input	1/h
heating_type	Heating-system category	-
cooling_type	Cooling-system category	-
heating_source_HSPF	Source heat-pump heating seasonal performance factor from ResStock	Btu/Wh
heating_source_efficiency	Source heating efficiency for non-heat-pump systems	%
cooling_source_SEER	Source cooling seasonal energy efficiency ratio from ResStock	Btu/Wh
heating_COP	Rated heating coefficient of performance used as the EnergyPlus input	-
cooling_COP	Rated cooling coefficient of performance used as the EnergyPlus input	-
case_weight	Statistical weight assigned to each unique configuration	cases

The case_weight field the number of analytical cases represented by each unique configuration within the probability-weighted 1200-case population. After applying these weights, the dataset represents 1000 single-family detached buildings and 200 single-family attached buildings. The weight can therefore be used when calculating population-level distributions or aggregated simulation results, whereas case_ID should be used to link the building descriptions to the corresponding simulation and output files.

Table 3 summarizes the 25 variables provided in each synthetic telemetry file (*.gz.parquet file). The outputs cover one full year at five-minute intervals and include outdoor weather, indoor and attic conditions, thermostat setpoints, air-exchange rates, and HVAC operation. HVAC variables include runtime fractions, heating and cooling rates, energy use, and supply-fan operation. Runtime fractions range from 0 to 1, where 0 indicates no operation and 1 indicates continuous operation during the reporting interval. Fig. 1 shows an example telemetry profile for a synthetic residential case in North Carolina, obtained from 001.gz.parquet.

**Table 3:** Synthetic telemetry output variables and units.

Category	Output variable	Unit
Time	Date/Time	—
Outdoor weather	Outdoor dry-bulb temperature	°C
	Outdoor dew-point temperature	°C
	Outdoor relative humidity	%
	Wind speed	m/s
	Direct solar radiation	W/m²
	Diffuse solar radiation	W/m²
Indoor air condition	Living-zone air temperature	°C
	Living-zone relative humidity	%
	Attic air temperature	°C
	Attic relative humidity	%
Thermostat settings	Heating setpoint temperature	°C
	Cooling setpoint temperature	°C
Air exchange	Infiltration air-change rate	ACH
	Mechanical ventilation rate	ACH
Heating operation	Heating-coil runtime fraction	0–1
	Heating-coil heating rate	W
	Heating-coil natural-gas energy	J
	Heating-coil electricity energy	J
Cooling operation	Cooling-coil runtime fraction	0–1
	Total cooling rate	W
	Sensible cooling rate	W
	Cooling-coil electricity energy	J
Fan operation	Supply-fan runtime fraction	0–1
	Supply-fan electricity rate	W

**Fig. 1:**
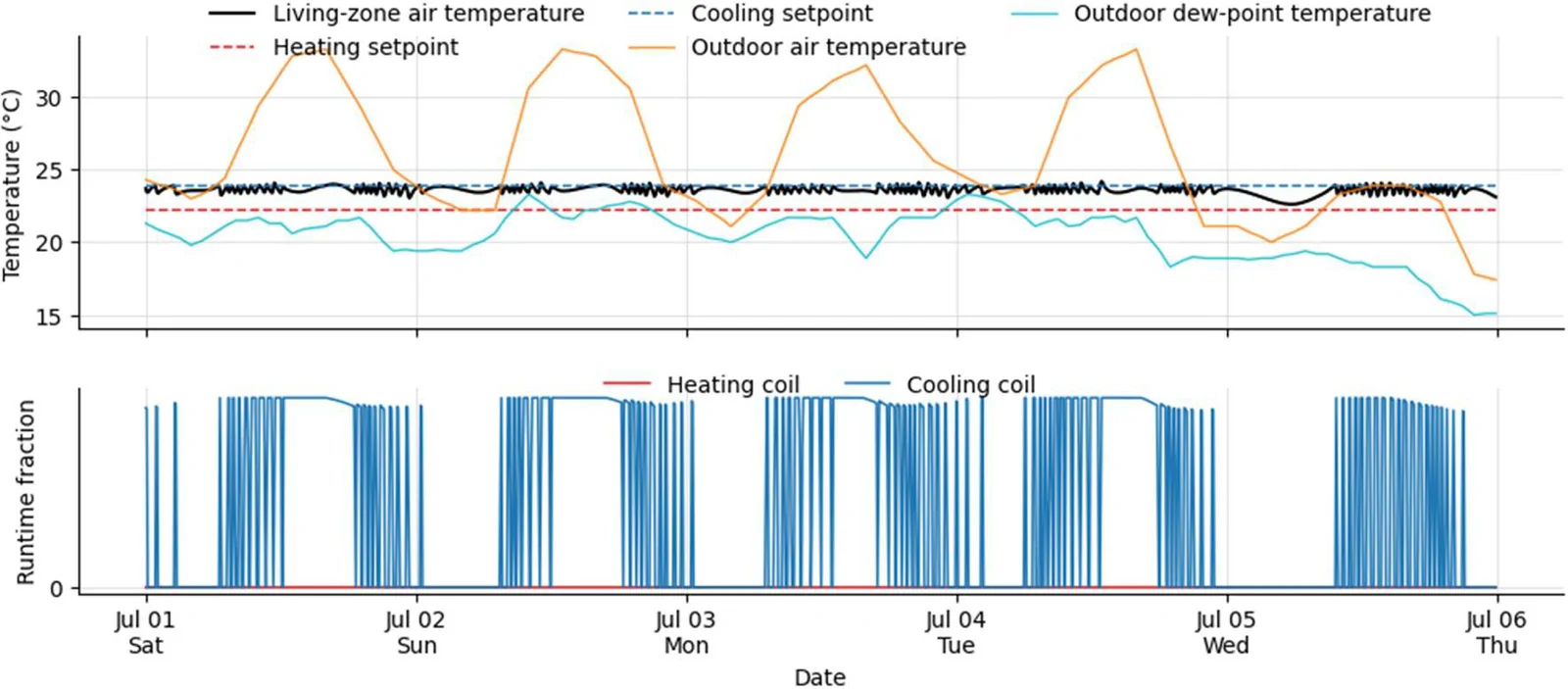
Example five-minute synthetic telemetry for July 1–5.

## 4 Experimental Design, Materials and Methods

Fig. 2 summarizes the workflow used to generate the dataset. Probability distributions from ecobee DYD metadata and ResStock were used to define representative residential configurations. The selected cases were converted into EnergyPlus models and simulated for one year at five-minute resolution. The simulation outputs were processed into synthetic telemetry files, and the resulting dataset was evaluated by comparing HVAC runtime patterns with ecobee field telemetry and by examining differences between detached and attached homes.

**Fig. 2:**
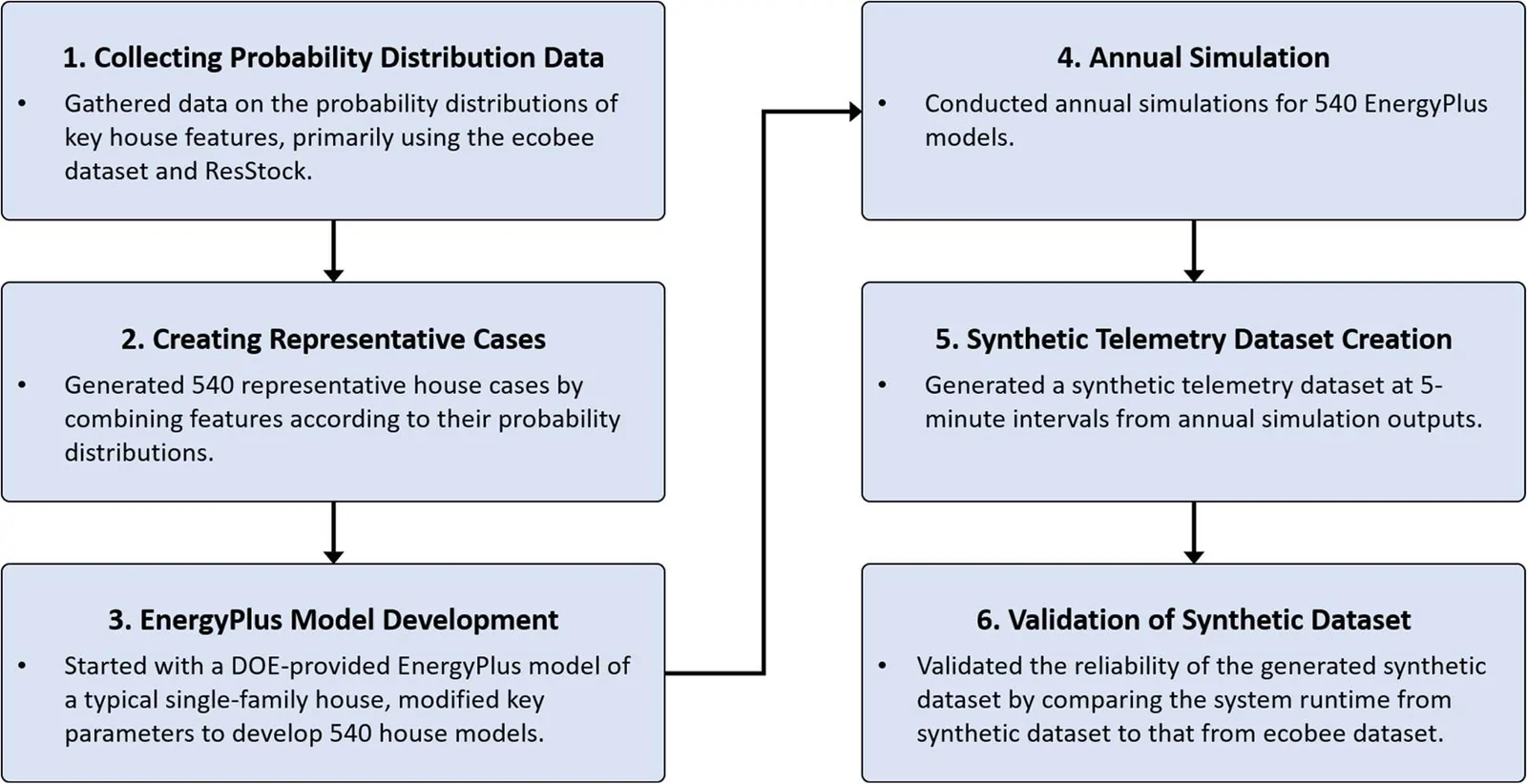
Workflow for synthetic residential model generation, EnergyPlus simulation, five-minute telemetry processing, and dataset evaluation.

### 4.1 Source data

The synthetic residential cases were generated from probability distributions of location, vintage, housing type, structural system, wall and ceiling insulation, window type, infiltration, HVAC configuration, and heating and cooling efficiency. ecobee DYD metadata, obtained in February 2022, were used to derive the distributions of location, construction vintage, housing type, and HVAC system configuration from a field-home sample of 4789 homes, including 3932 detached and 857 attached homes. Fig. 3, Fig. 4, Fig. 5 shows representative examples of these ecobee-derived distributions. Alaska and Hawaii were excluded because both the ecobee DYD and ResStock datasets contained insufficient housing-stock information for these states.

**Fig. 3:**
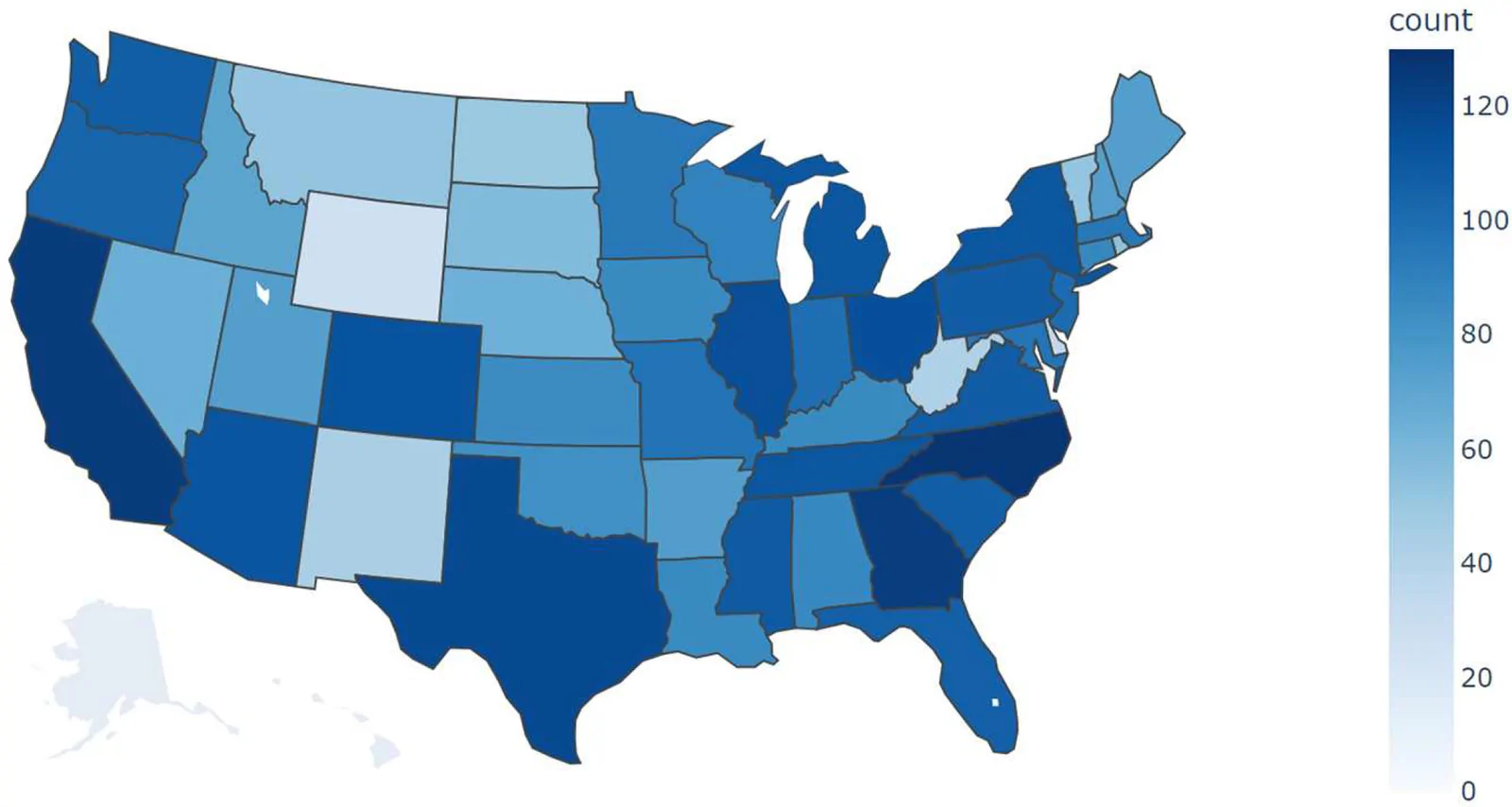
Distribution of ecobee sample by location for detached homes.

**Fig. 4:**
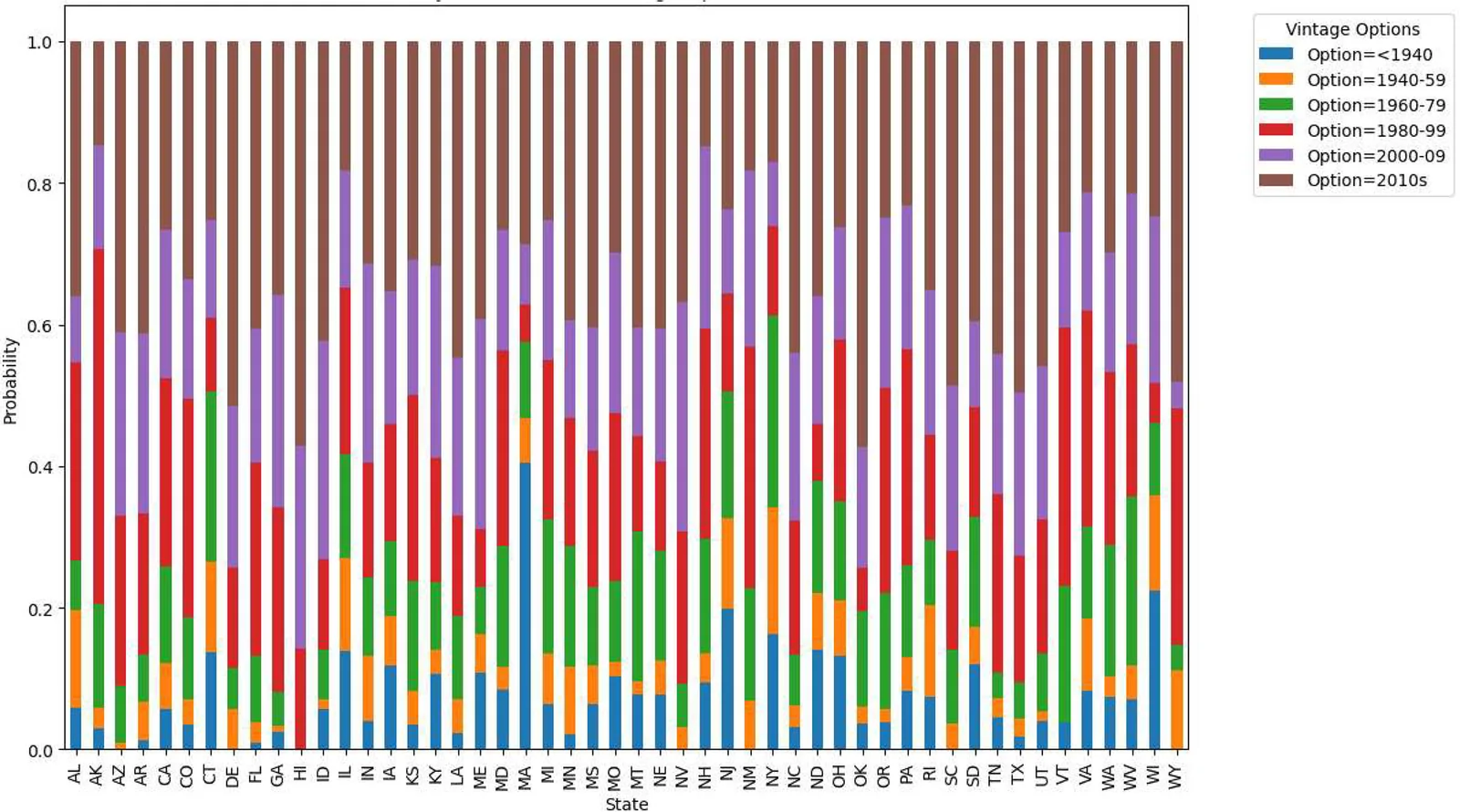
Distribution of ecobee sample by vintage for detached homes.

**Fig. 5:**
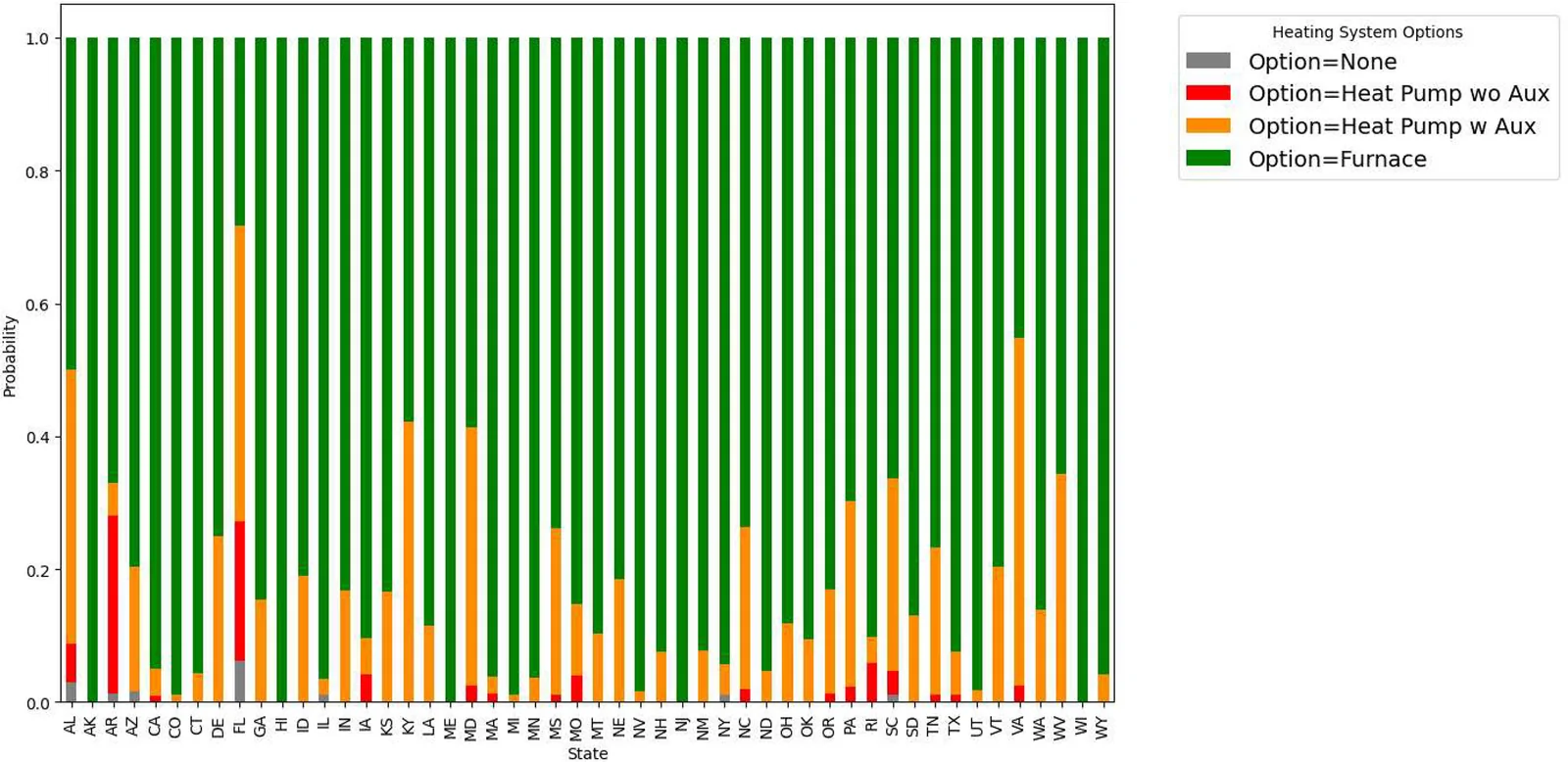
Distribution of ecobee sample by system type for detached homes.

ResStock supplied attributes unavailable in the ecobee metadata, including structural system, insulation, window properties, infiltration, equipment efficiency, and horizontal unit location for attached homes. The building-energy models were based on the 2021 ResStock End-Use Load Profiles release (resstock_tmy3_release_1). Attribute probabilities retained their documented dependencies [2]; for example, wall-insulation probabilities varied by location, vintage, and structure. Table 4 summarizes the possible values for each attribute, the factors on which their probabilities depend, and the sources of the distributions.

**Table 4:** Building attributes, options, conditioning variables, and probability-distribution sources.

Attributes	Options	Conditioned on	Sources
Location	48 states (excluding Alaska and Hawaii)	-	ecobee
Horizontal unit location	Middle or side unit (attached homes only)	Housing type	ResStock
Vintage bin	Pre-1940; 1940–59; 1960–79; 1980–99; 2000–09; 2010s	Location	ecobee
Structure	Steel frame; wood frame; concrete; brick	Location, vintage	ResStock
Wall insulation	Uninsulated; R-7; R-11; R-15; R-19	Location, vintage, structure	ResStock
Ceiling insulation	Uninsulated; R-7; R-13; R-19; R-30; R-38; R-49	Location, vintage	ResStock
Window type	Single clear; double clear; double low-e; triple low-e	Location, vintage	ResStock
Infiltration	1–50 ACH50	Location, vintage	ResStock
HVAC system	Heat-pump and furnace combinations, with/without cooling or auxiliary heat	Location, vintage	ecobee
Heating efficiency	Heat pump: HSPF 6.2, 7.7, 8.5; furnace: 60%, 76%, 80%, 92.5%	System type	ResStock
Cooling efficiency	SEER 8, 10, 13, 15	Vintage	ResStock

Probability distributions derived from ecobee DYD and ResStock are not redistributed in the repository because access and redistribution are governed by the respective data providers; researchers seeking these source data should contact the original providers directly.

### 4.2 Probability-based synthetic residential case generation

All possible combinations of categorical attributes were constructed probabilistically. Direct enumeration of every numerical option in Table 4 would have produced approximately 3.53 billion combinations. Conversely, assigning only the most probable option for each attribute would have substantially reduced the diversity of the represented residential stock. Therefore, a hybrid approach, illustrated in Fig. 6, was used in which major categorical attributes (location, vintage bin, structure type, horizontal unit location for attached houses, window type, and HVAC system type) were enumerated, while continuous characteristics and attributes with numerous numerical options (wall insulation, ceiling insulation, infiltration, heating efficiency, and cooling efficiency) were represented by conditional probability-weighted averages. This procedure reduced the model space while preserving prevalent combinations and variation in major building and system characteristics.

**Fig. 6:**
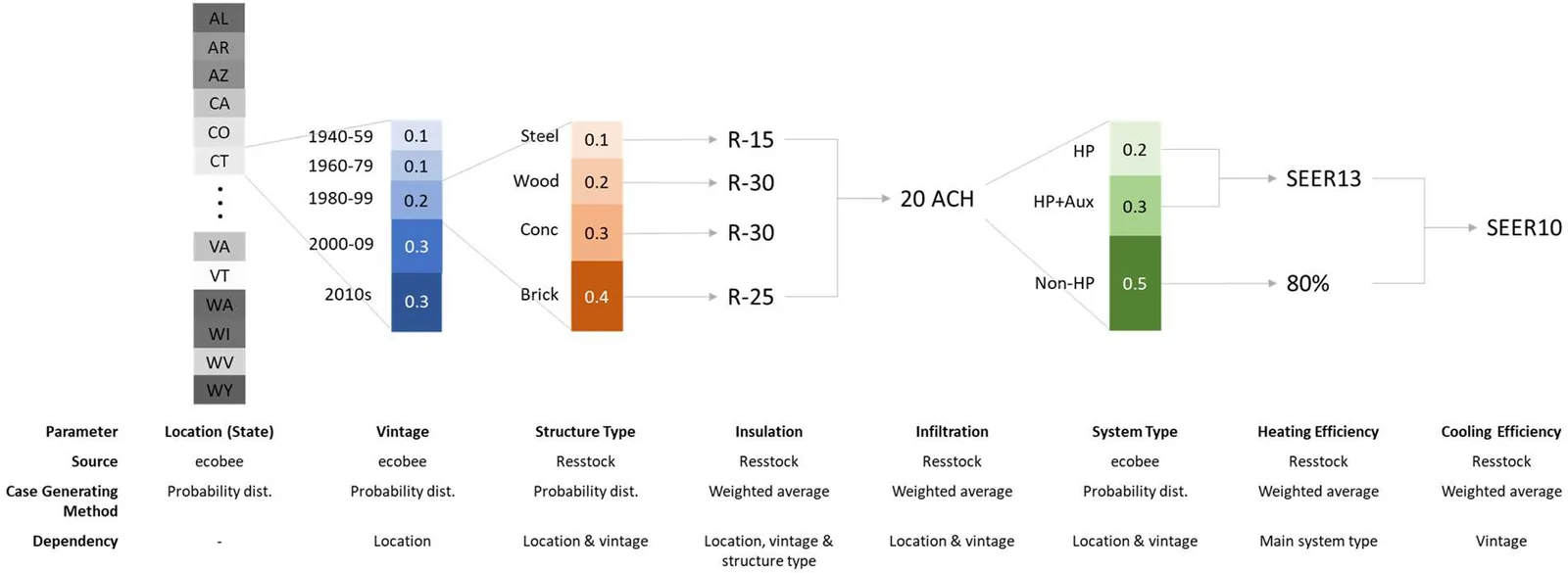
Hybrid combination and weighted-average approach used to generate synthetic residential cases.

The probability-weighted values should be interpreted as conditional representative values rather than probabilistic samples from the full underlying distributions. As a result, this treatment reduces within-group variability and does not preserve the full variance and covariance of the averaged physical attributes. This limitation should be considered in applications requiring detailed building-to-building heterogeneity or uncertainty propagation.

The joint probability of each candidate configuration was calculated by multiplying its geographic probability by the relevant conditional attribute probabilities, with the conditioning relationships determined from the source datasets and summarized in Table 4. In simplified form:

(1) $$P_{i}=P\left(L_{i}\right)P\left(V_{i}|L_{i}\right)P\left(W_{i}|L_{i},V_{i}\right)P\left(G_{i}|L_{i},V_{i}\right)P\left(H_{i}|L_{i},V_{i}\right)$$

where L,V,W,G and H denote location, vintage, structure type, window type, and HVAC system type, respectively.

Continuous characteristics and attributes with numerous numerical options were represented using conditional probability-weighted averages. For a characteristic x with possible values $x_{j}$ and corresponding conditional probabilities $p_{j}$, the representative value was calculated as:

(2) $$\bar{x}=\sum_{j=1}^{n}p_{j}x_{j},\;\;\sum_{j=1}^{n}p_{j}=1$$

For example, probabilities of 0.10, 0.20, 0.60, and 0.10 associated with insulation values of R-7, R-11, R-13, and R-15, respectively, produce a representative value of:

(3) $$\bar{R}=\left(0.10\;\times \;7\right)+\left(0.20\;\times \;11\right)+\left(0.60\;\times \;13\right)+\left(0.10\;\times \;15\right)=12.2$$

This approach was applied to wall and ceiling insulation levels, infiltration rates, and heating and cooling system efficiencies.

The hybrid categorical-enumeration and weighted-average procedure produced 1602 and 1480 candidate configurations for detached and attached homes, respectively. The reduced analytical stock was first defined as 1000 detached and 200 attached homes to approximately reflect the predominance of detached homes in the source ecobee DYD population (approximately 82% detached and 18% attached). Within each housing type, candidate configurations were ranked by joint probability, and the 360 highest-probability detached and 180 highest-probability attached configurations were retained as unique simulations. Their normalized probabilities were then converted to case weights, such that the 360 detached simulations represented 1000 analytical cases and the 180 attached simulations represented 200 analytical cases.

Fig. 7 shows the cumulative normalized probability as a function of configuration rank for each housing type. The retained 360 detached configurations accounted for 96.7% of the cumulative probability mass of all detached-home candidate configurations, whereas the retained 180 attached configurations accounted for 75.0% of the cumulative probability mass of all attached-home candidate configurations. Thus, the reduction substantially decreased the number of configurations requiring simulation while retaining most of the probability mass, particularly for detached homes. The lower probability coverage of attached homes should be considered when using the dataset to characterize the diversity of attached houses.

**Fig. 7:**
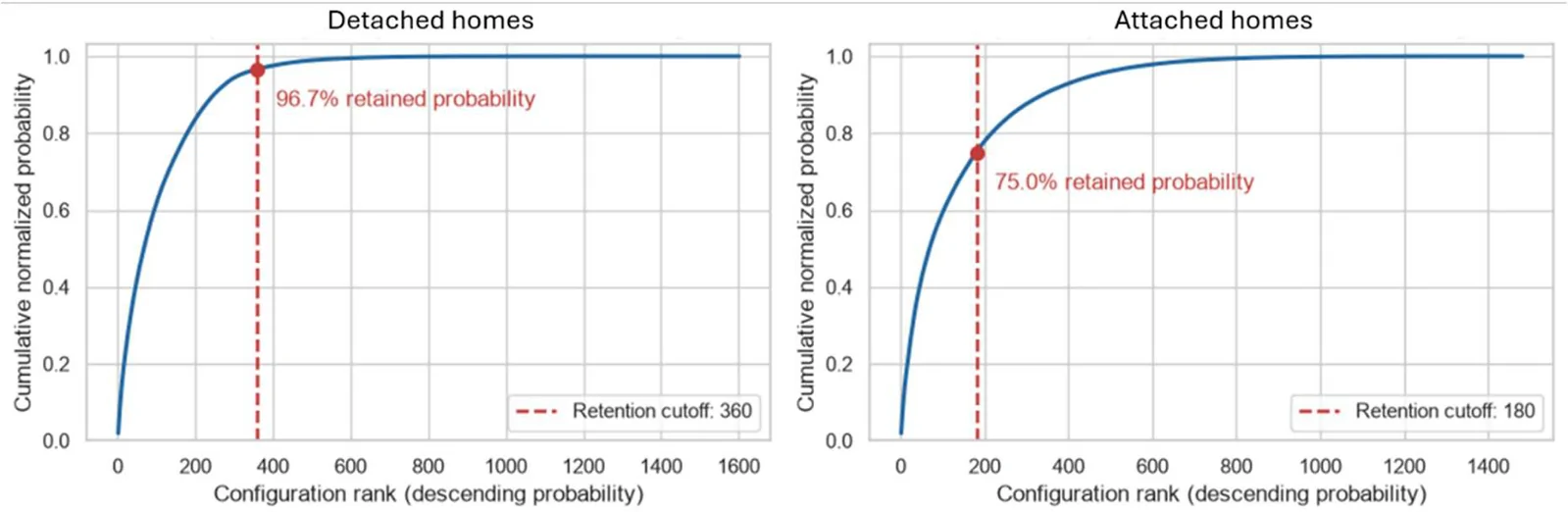
Cumulative normalized probability of candidate configurations ranked by joint probability for detached (left) and attached (right) homes.

Overall, the 540 unique simulations represent a probability-weighted analytical population of 1200 residential cases. Each model was simulated once, while its case weight reflects the relative prevalence of its geographic, building-envelope, and HVAC characteristics in subsequent stock-level analyses.

### 4.3 EnergyPlus model generation and annual simulation

EnergyPlus version 9.5 was used to compile and simulate the synthetic residential cases. The U.S. Department of Energy (DOE) residential prototype model [4] was used as the detached-house baseline. It represents a two-story single-family detached house with an attic, 2377 ft² of conditioned floor area, and a slab-on-grade foundation without a basement. The detached-house base model geometry is illustrated in Fig. 8.

**Fig. 8:**
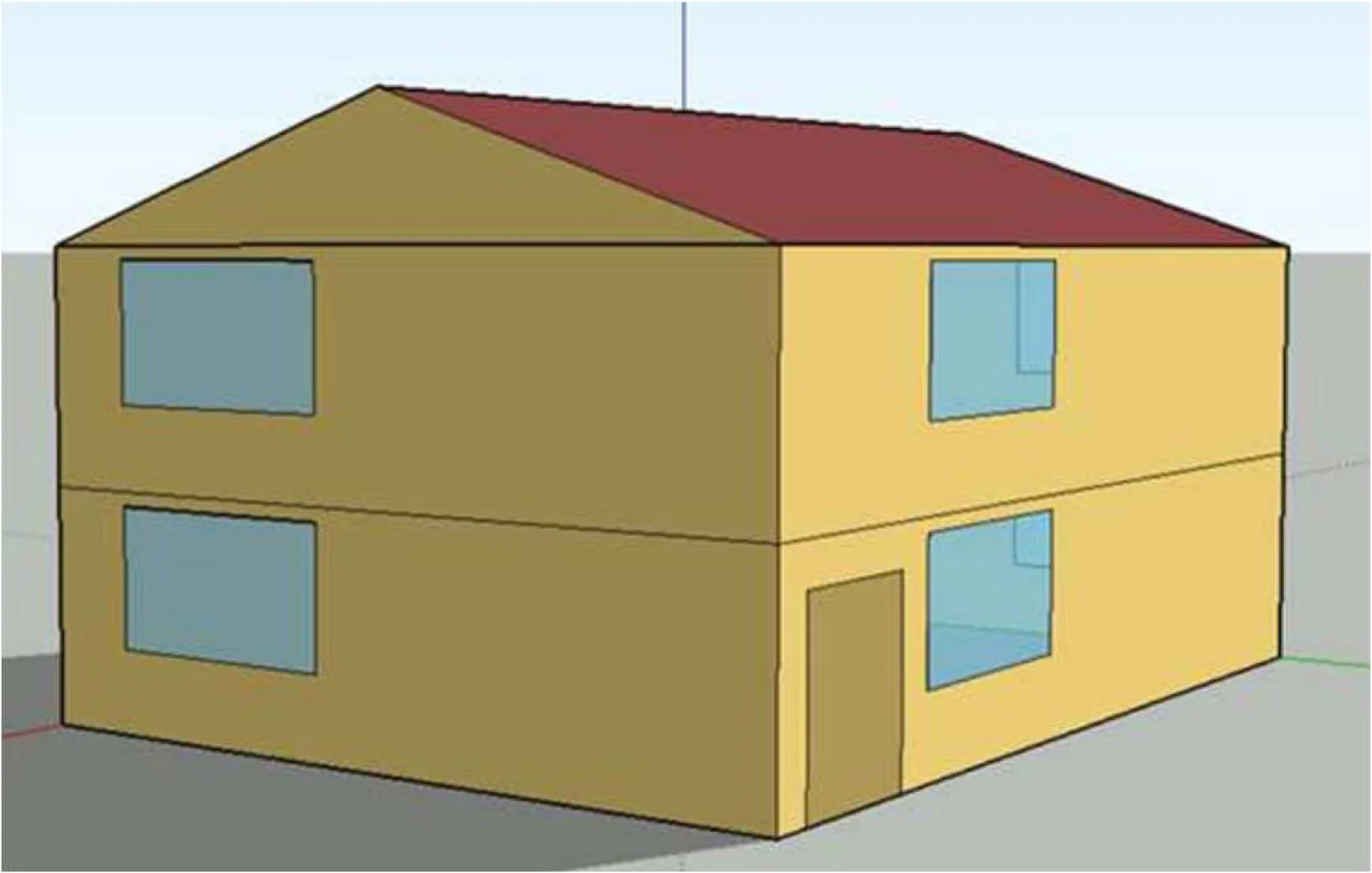
Detached-house base geometry.

Because the DOE prototypes do not provide a representative attached-house geometry for this purpose, custom two-story attached base models were created. The 2077 ft² floor area was derived from the average floor area of attached homes in the ecobee DYD metadata. Because the ecobee metadata do not provide detailed geometric information such as foundation type, attic configuration, number of stories, aspect ratio, or window area, these characteristics were defined using average or modal conditions from ResStock. Accordingly, both attached models were configured as two-story, slab-on-grade buildings with no attic, a 1:1.8 width-to-depth ratio (24 ft × 43.3 ft), and 84.42 ft² of window area. Because no attic or basement is included, the modeled gross floor area is equivalent to the conditioned floor area.

The side-unit model has one adiabatic shared wall and represents a semi-detached home or an end unit of a rowhouse. The middle-unit model has two adiabatic shared walls and represents an interior rowhouse unit. Unit location was assigned using ResStock probabilities. The attached-house base model geometry is illustrated in Fig. 9.

**Fig. 9:**
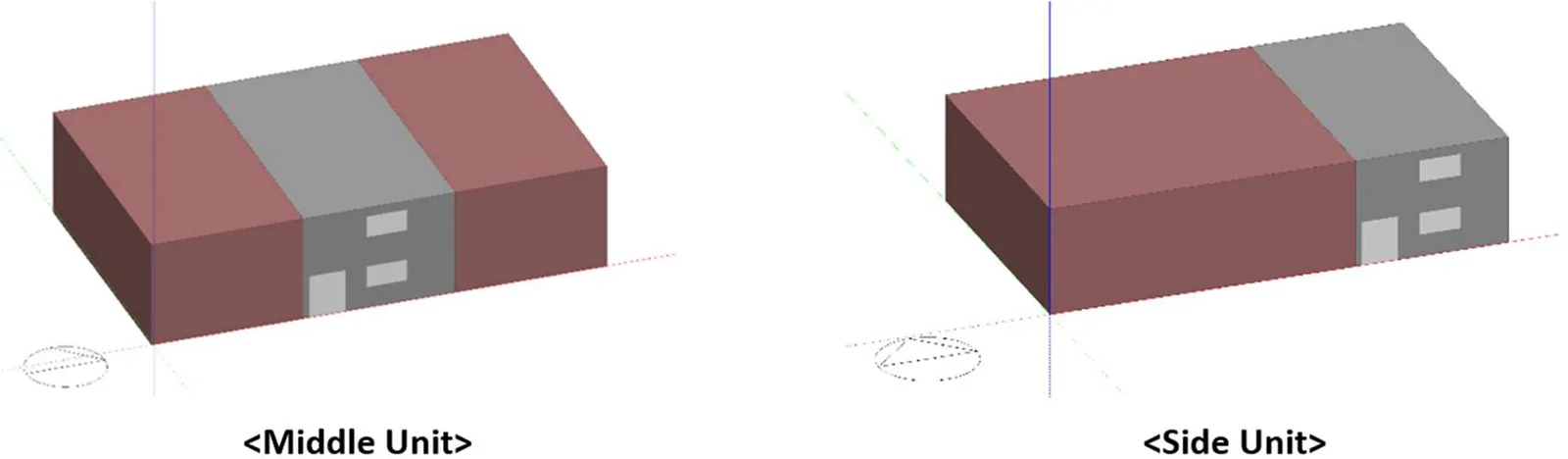
Attached-house base geometries: middle unit with two shared walls and side unit with one shared wall.

For each state, the city containing the largest number of ecobee DYD homes was selected as the simulation location. The corresponding TMY, TMY2, or TMY3 weather file was obtained from the EnergyPlus weather database [5]. The weather station locations selected for the 48 states and their ASHRAE climate-zone classifications are summarized in Table 5. When no weather file was available for the representative city, an available weather station within 10 miles of the representative city was selected. If no weather station was available within 10 miles, the city with the next-largest number of ecobee DYD homes in the state was considered, and the same weather-file selection procedure was repeated. The representative ecobee city, ecobee home count, selected weather station, and corresponding weather file for each state are provided in weather_data_summary.csv. The 13 climate zones are represented by selected locations and do not capture the full range of conditions within each zone. ecobee metadata indicates whether the main heating system is a heat pump and whether auxiliary heat is present, but does not identify every component. For non-heat-pump systems, a natural-gas furnace was assumed because it is the most prevalent non-heat-pump heating system in U.S. single-family homes according to the 2024 Residential Energy Consumption Survey [6]. For heat-pump systems with auxiliary heating, electric resistance heater was assumed.

**Table 5:** Weather stations and ASHRAE climate zones represented in the synthetic dataset.

State	Weather Station	ASHRAE Climate Zone	State	Weather Station	ASHRAE Climate Zone
Alabama	Huntsville International Airport-Jones Field	3A	North Carolina	Raleigh-Durham International Airport	3A
Arkansas	Little Rock-Adams Field	3A	North Dakota	Fargo-Hector International Airport	7
Arizona	Luke Air Force Base	2B	Nebraska	Holdrege-Brewster Field	5A
California	Sacramento Executive Airport	3C	New Hampshire	Manchester Municipal Airport	5A
Colorado	Colorado Springs	5B	New Jersey	Newark International Airport	4A
Connecticut	Hartford-Brainard Field	4A	New Mexico	Truth or Consequences Municipal Airport	3B
Delaware	Dover Air Force Base	4A	Nevada	Reno-Tahoe International Airport	5B
Florida	Jacksonville Naval Air Station	2A	New York	Buffalo	5A
Georgia	Marietta-Dobbins Air Force Base	4A	Ohio	Ohio State University Airport	4A
Iowa	Clinton Municipal Airport	5A	Oklahoma	Tulsa International Airport	3A
Idaho	Idaho Falls-Fanning Field	6B	Oregon	Portland-Hillsboro Airport	4C
Illinois	Chicago O’Hare International Airport	5A	Pennsylvania	Pittsburgh	5A
Indiana	Indianapolis International Airport	5A	Rhode Island	Pawtucket Airport	5A
Kansas	Olathe-Johnson County Executive Airport	4A	South Carolina	Greenville Downtown Airport	3A
Kentucky	Lexington	4A	South Dakota	Sioux Falls-Foss Field	6A
Louisiana	Baton Rouge	2A	Tennessee	Memphis International Airport	3A
Massachusetts	Boston Logan International Airport	5A	Texas	Dallas-Addison Airport	2B
Maryland	Baltimore/Washington International Airport	4A	Utah	Salt Lake City International Airport	4B
Maine	Portland International Jetport	6A	Virginia	Ronald Reagan Washington National Airport	4A
Michigan	Detroit City Airport	5A	Vermont	Burlington International Airport	5A
Minnesota	Minneapolis-Saint Paul International Airport	6A	Washington	Seattle-Boeing Field	4C
Missouri	Spirit of St. Louis Airport	4A	Wisconsin	Milwaukee	6A
Mississippi	Greenville Municipal Airport	3A	West Virginia	Charleston	4A
Montana	Bozeman-Gallatin Field	6B	Wyoming	Casper-Natrona County International Airport	6B

HVAC system capacities were determined using the EnergyPlus autosizing [7] procedure because realistic case-specific equipment-size information was not available from the source datasets. Accordingly, the resulting capacities represent simulation-based design sizes rather than measured or field-observed installed capacities. For heat pump cases, rated coefficient of performance (COP) for heating and cooling were obtained from corresponding ResStock models based on HSPF and SEER, respectively. Because the system-efficiency distributions were derived from ResStock, ResStock-based COP values were used to maintain consistency with the source data. For efficiency levels not explicitly represented in ResStock, COP values were linearly interpolated between the nearest available ratings. This approach also avoids assuming a unique direct conversion from seasonal SEER and HSPF ratings to the rated COP inputs required by EnergyPlus.

To represent residential cycling more realistically, the default EnergyPlus part-load formulation [7] was replaced with the HVAC on/off Energy Management System controller developed by Cetin et al. [8]. The controller overrides the default part-load-curve mechanism, monitors zone temperature and thermostat setpoints, and switches the HVAC among heating, cooling, and off modes using a ± 0.5 °C deadband. When active, the corresponding coil operates at full output; otherwise, supply airflow is set to zero.Standard DOE prototype setpoints were 22.2 °C for heating and 23.9 °C for cooling.

ACH50 distributions were derived from ResStock. For each airtightness and climate condition, corresponding ResStock models were simulated using their leakage-area-based infiltration formulation, and the resulting annual mean infiltration air change rate (1/hr) was used as the EnergyPlus input for the synthetic model. This simulation-based mapping was used instead of a fixed ACH50-to-natural-infiltration conversion [9] to preserve the climate-dependent infiltration assumptions represented in ResStock. Both the original ACH50 values and the resulting infiltration rates are reported in case_description.csv for reproducibility.

Occupancy and operational assumptions followed the DOE residential prototype and were held constant across synthetic cases for modeling simplicity. Each dwelling was modeled with three occupants using a fixed daily occupancy profile. This simplification reduces occupant-behavior variability and likely contributes to the convergence of synthetic heating and cooling balance temperatures relative to the ecobee field data. Accordingly, the dataset does not capture the full range of occupant-driven operational behavior and should be used with caution for applications focused on occupant behavior or demand response.

Each of the 540 synthetic EnergyPlus models was simulated for a full year, with outputs recorded at five-minute intervals and stored in compressed Parquet format (*.gz.parquet). The selected outputs include thermostat variables, such as indoor conditions, temperature setpoints, and HVAC runtime, as well as ground-truth infiltration rates and energy-use variables that are generally unavailable from field thermostats.

To quantitatively assess how well the reduced set of 540 configurations preserved the distributions of the source population, the probability-weighted marginal distributions of four principal categorical characteristics (state, housing type, construction vintage, and HVAC system type) were compared with the corresponding distributions derived from the ecobee DYD data (Fig. 10). Overall, the reduced synthetic stock preserved the source distributions well for state and housing type, with maximum absolute percentage-point differences of 0.92 and 1.29 percentage points, respectively. The vintage distribution showed a larger but still moderate maximum difference of 6.77 percentage points. The largest deviation was observed for HVAC system type, with a maximum difference of 13.85 percentage points for homes using a non-heat-pump heating system with air-conditioning cooling. This deviation reflects the reduction of the original candidate space to the highest-probability configurations and indicates that the reduced stock preserves the dominant marginal characteristics of the source population more closely than some less frequent combinations. Accordingly, the 540 cases are intended as a probability-informed reduced synthetic stock rather than a complete statistical representation of the U.S. residential building population.

**Fig. 10:**
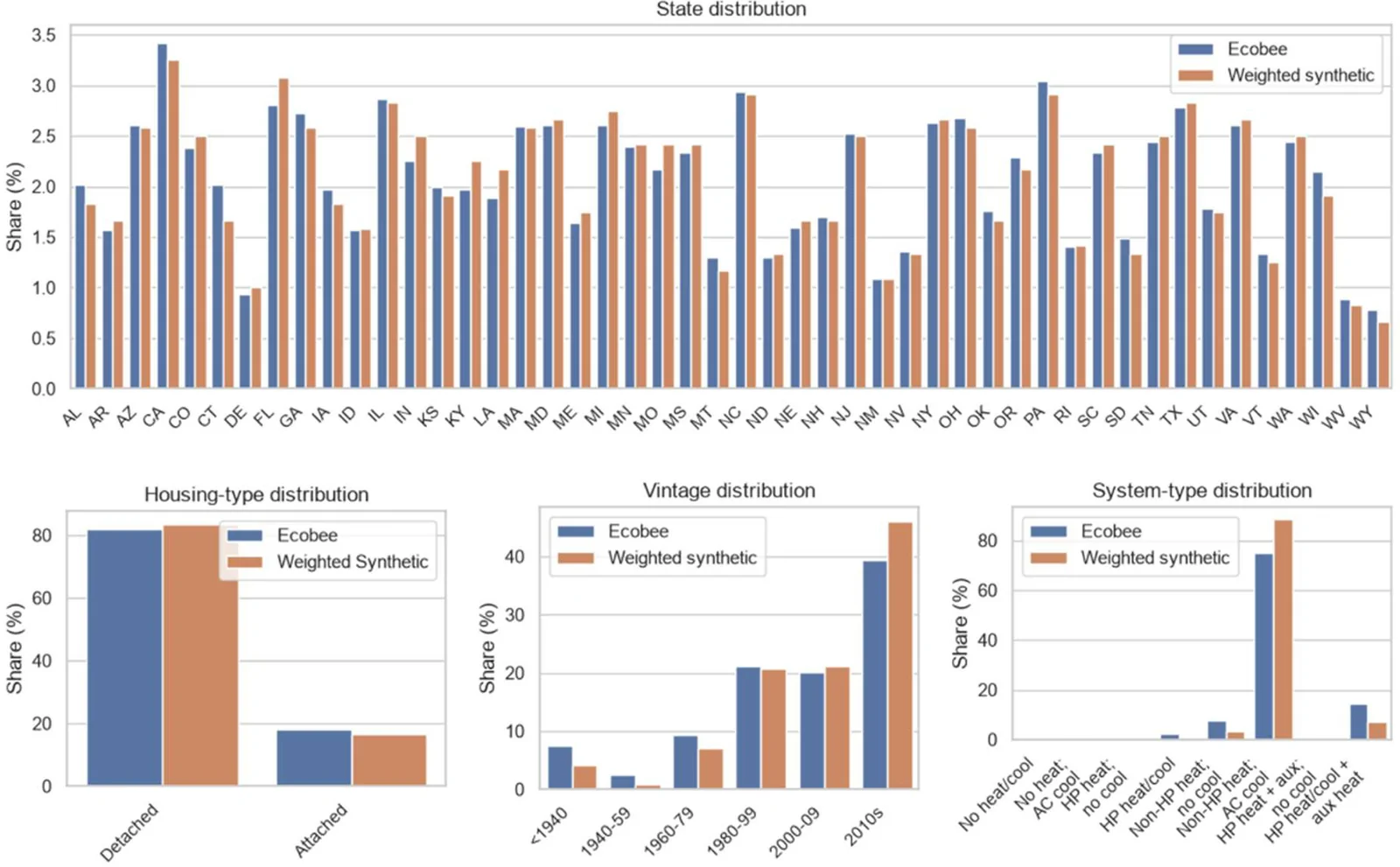
Comparison of marginal distributions between the source ecobee DYD population and the probability-weighted 540-case synthetic stock for state, housing type, construction vintage, and HVAC system type.

### 4.4 Validation of HVAC-runtime patterns against ecobee field telemetry

Synthetic-data realism was evaluated specifically with respect to the relationship between outdoor temperature and HVAC runtime using a four-parameter change-point model (CPM) following the inverse-modeling procedure of Kissock et al. [10]. Similar HVAC runtime-temperature relationships and associated CPM parameters have previously been used to characterize HVAC operation across a large sample of U.S. homes using ecobee data [11]. Daily cumulative HVAC runtime Y was modeled as a function of daily mean outdoor-air temperature X:

(4) $$Y=\beta_{1}{\left(X-\beta_{3}\right)}^{-}+\beta_{2}{\left(X-\beta_{4}\right)}^{+}$$

Here, $\beta_{1}$ and $\beta_{2}$ are the heating and cooling runtime slopes, and $\beta_{3}$ and $\beta_{4}$ are the heating and cooling balance-point temperatures. Candidate heating and cooling balance points were optimized by grid search. For each candidate pair, piecewise linear regression estimated the slopes, and the parameter set with the highest coefficient of determination $\left(R^{2}\right)$ was retained. The Python implementation used for CPM fitting, including change-point optimization and parameter estimation, is publicly available in the accompanying repository [12].

CPM parameters were aggregated across 13 ASHRAE climate zones. The EnergyPlus comparison used the weighted 1200-house analytical sample. The ecobee telemetry data used for validation covers July 2020 through June 2021. Of the 4789 ecobee homes initially considered, homes were required to have at least 255 valid days out of the 365-day analysis period (70% coverage), leaving 3908 homes. This filtering procedure may introduce selection bias by preferentially retaining homes with more complete telemetry records.

All four CPM parameters from EnergyPlus and ecobee were compared across climate zones using RMSE and coefficient of variation of RMSE (CV(RMSE)). CV(RMSE) normalizes RMSE by the corresponding mean ecobee value and expresses the error as a percentage.

Table 6 compares the CPM parameters derived from the ecobee and EnergyPlus datasets for the full sample. The CV(RMSE) values ranged from 10.9% to 23.1%, indicating reasonable aggregate agreement between the synthetic EnergyPlus results and ecobee telemetry. ASHRAE Guideline 14–2014 specifies CV(RMSE) thresholds of 15% for monthly calibration and 30% for hourly calibration but does not establish a threshold for daily data [13]. Therefore, values reported in prior daily-resolution studies, including approximately 20–25% [14,15], are used here only as contextual references rather than formal acceptance criteria, and all calculated values were below 25%.

**Table 6:** Comparison of CPM parameters between ecobee telemetry and synthetic telemetry.

CPM parameter	ecobee mean	EnergyPlus mean	RMSE	CV(RMSE)
Heating slope (h/ °C)	−0.39	−0.42	0.09	23.1%
Cooling slope (h/ °C)	0.85	0.84	0.09	10.9%
Heating balance temperature ( °C)	14.20	15.62	1.62	11.4%
Cooling balance temperature ( °C)	16.50	15.62	1.96	11.9%

EnergyPlus also generally reproduced the climate-zone patterns observed in the ecobee data, as shown in Fig. 11. Both datasets showed stronger heating responses in colder climate zones, whereas cooling responses varied more across warm and mixed climates. Although differences in magnitude occurred in some zones, the broad overlap between the distributions suggests that the synthetic stock captures much of the climate-dependent patterns in the temperature-runtime relationship.

**Fig. 11:**
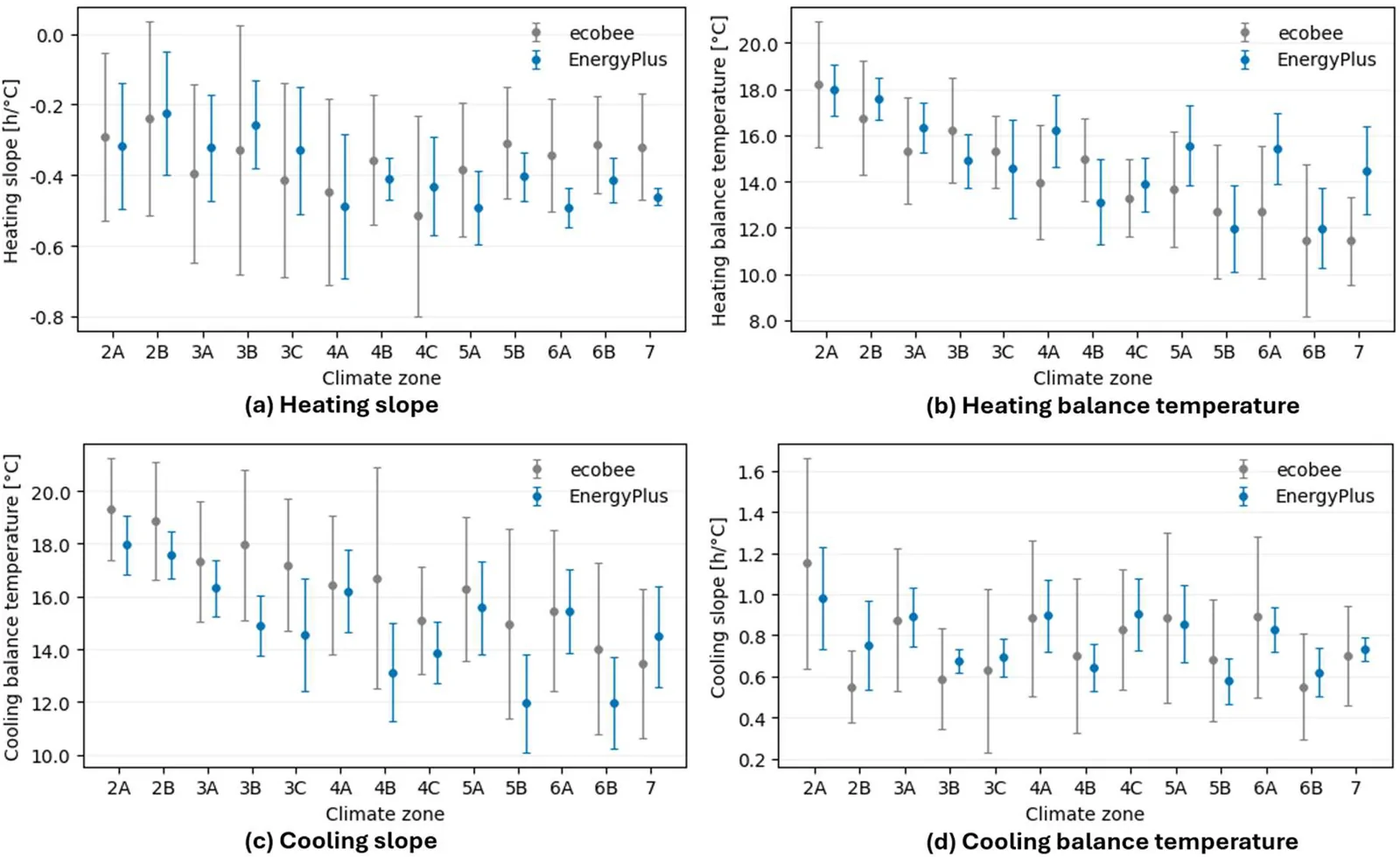
Change point model parameters by climate zone: ecobee vs. EnergyPlus (Error bars show the 5th and 95th percentile range.).

Fig. 12, Fig. 13 compare the CPM curves derived from ecobee and synthetic telemetry at the national level and across climate zones, respectively. The synthetic telemetry generally follows the heating and cooling response patterns observed in the ecobee data, indicating acceptable agreement, while the wider 5th-95th percentile ranges for ecobee reflect greater variability among actual homes. The most notable discrepancy was in the balance temperatures. As shown in Fig. 12, ecobee exhibited distinct mean heating and cooling balance temperatures of 14.2 °C and 16.5 °C, respectively, whereas EnergyPlus produced nearly identical values of approximately 15.6 °C for both. This difference may be attributable to the standardized thermostat setpoints adopted from the DOE prototype models (22.2 °C for heating, 23.9 °C for cooling). In contrast, actual thermostat setpoints in the ecobee DYD dataset vary among homes and over time. Differences in internal heat gains and occupant behavior may further shift the observed balance temperatures.

**Fig. 12:**
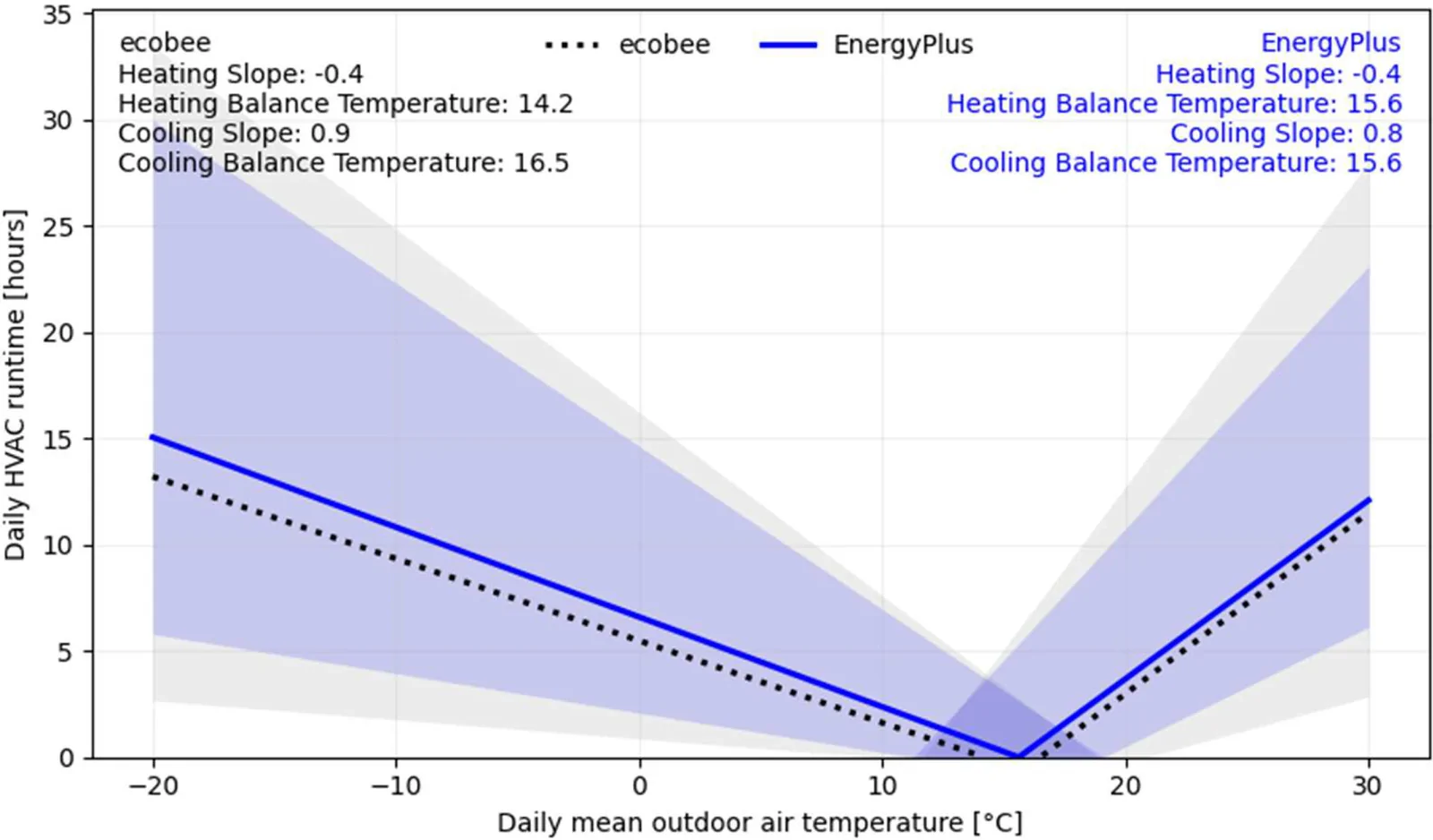
Whole-U.S. comparison of CPM curves derived from ecobee and synthetic telemetry (The shaded regions represent the 5th–95th percentile ranges of the CPM curves.).

**Fig. 13:**
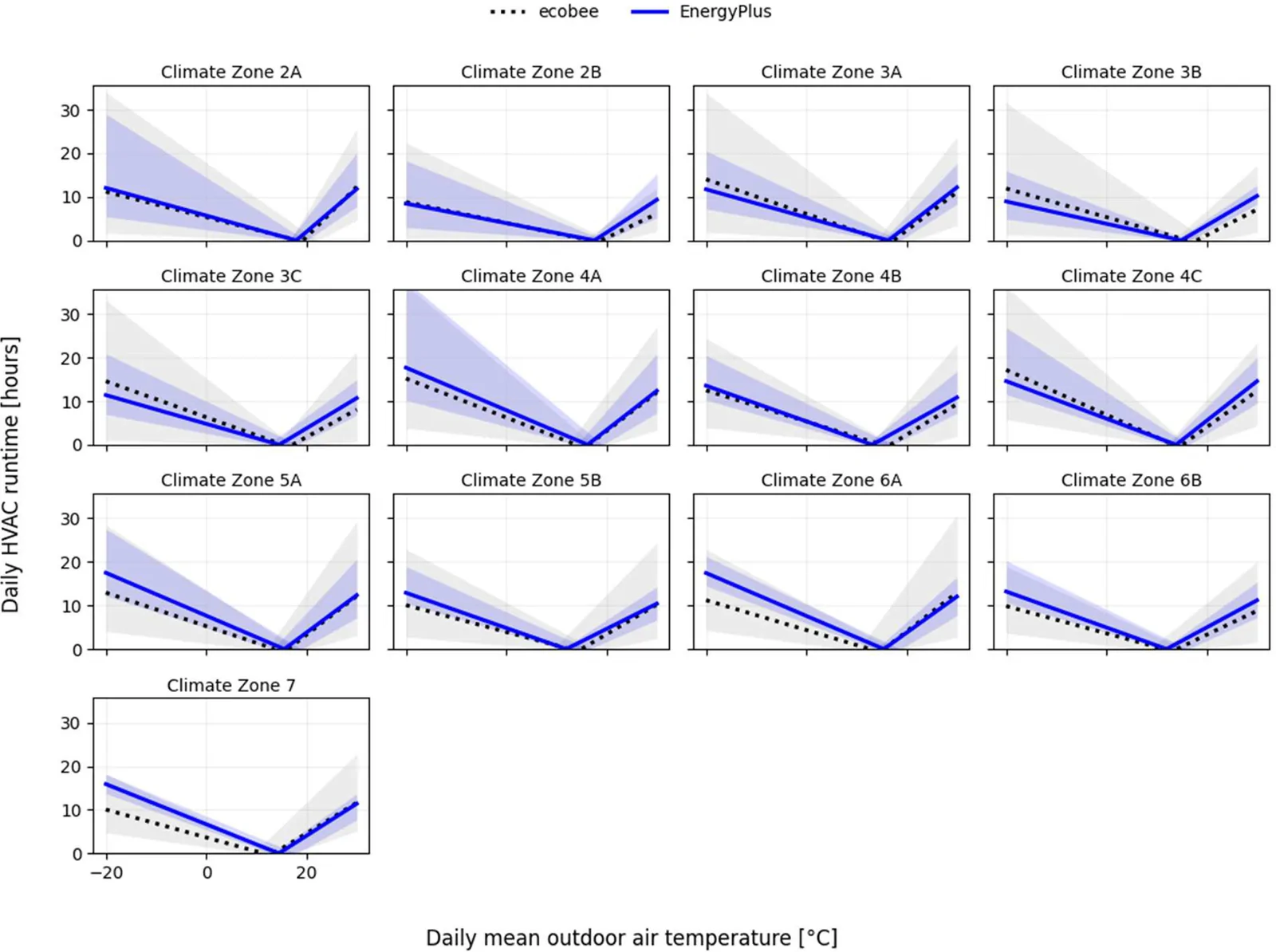
Comparison of CPM curves derived from ecobee and synthetic telemetry by climate zones (The shaded regions represent the 5th–95th percentile ranges of the CPM curves.).

Overall, the synthetic models reproduce the temperature-runtime relationship and its climate- dependent patterns observed in ecobee field telemetry, while differences in balance-point temperatures highlight the limitations introduced by standardized thermostat and occupancy assumptions. Climate-zone-level means, standard deviations, and sample counts for all four CPM parameters in both datasets are provided in cpm_climate_zone_summary.csv.

## Limitations

The dataset is a synthetic representation of the U.S. residential building stock and does not contain calibrated models of individual homes. The ecobee DYD metadata were obtained from participating smart-thermostat users and may not fully represent the national housing population. Retaining high-probability configurations excludes some uncommon building and HVAC characteristics, while probability-weighted averaging of continuous or high-option attributes reduces within-category variability and may produce more average parameter combinations than occur in individual homes.

Each state is represented by one weather station and a typical-year weather file, so the dataset does not capture within-state variability, local microclimates, or interannual weather variation. Fixed base geometries limit variation in floor area, layout, and envelope configuration. The dataset includes only single-family detached and attached homes, excluding multifamily and mobile homes. Standardized occupancy, internal gains, and HVAC operation also limit representation of occupant behavior and field system operation, particularly for applications requiring realistic behavioral variability.

Accordingly, the dataset is best suited for benchmarking, comparative modeling, and method development rather than precise prediction of individual-building performance. Applications involving retrofit assessment, HVAC sizing, uncertainty analysis, or national-scale inference should account for these simplified physical, operational, and weather assumptions.

## Ethics Statement

The authors have read and followed the ethical requirements for publication in Data in Brief. This work used de-identified secondary building metadata and synthetic simulation data and did not involve human subjects, animal experiments, or data collected from social media platforms.

## Credit Author Statement

**You-Jeong Kim**: Conceptualization, Methodology, Data Curation, Validation, Writing - Original Draft. **Yun Kyu Yi**: Methodology, Supervision, Funding acquisition, Writing - Review & Editing.

## References

[1] ecobee Inc., Donate Your Data, ecobee Inc., Toronto, Canada, Available at: https://www.ecobee.com/en-us/donate-your-data/ (accessed 19 March 2026).

[2] National Renewable Energy Laboratory. *ResStock: Housing Characteristics Probability Distributions*. National Renewable Energy Laboratory, Golden, CO, USA, GitHub repository. Available at: https://github.com/NatLabRockies/resstock/tree/develop/project_national/housing_characteristics (Accessed 20 November 2023).

[3] Kim Y.-J., Yi Y.K. (2026).

[4] Kneifel J. (2012).

[5] U.S. Department of Energy (2026). https://energyplus.net/weather.

[6] U.S. Energy Information Administration (2024). https://www.eia.gov/consumption/residential/data/2024/.

[7] U.S. Department of Energy (2021).

[8] Cetin K.S., Fathollahzadeh M.H., Kunwar N., Do H., Tabares-Velasco P.C. (2019). Development and validation of an HVAC on/off controller in EnergyPlus for energy simulation of residential and small commercial buildings. Energy Build..

[9] Sherman M.H. (1987). Estimation of infiltration from leakage and climate indicators. Energy Build..

[10] Kissock J.K., Haberl J.S., Claridge D.E. (2002). https://oaktrust.library.tamu.edu/items/e7e6e00f-e65d-45f8-bbb5-e1a2be10bcd1.

[11] Kim Y.J., Waegel A., Hakkarainen M., Yi Y.K., Braham W. (2025). Understanding HVAC system runtime of US homes: an energy signature analysis using smart thermostat data. Build. Simul..

[12] Kim Y.-J. (2026). https://github.com/yjkim9411/residential_hvac_cpm.

[13] American Society of Heating, Refrigerating and Air-Conditioning Engineers (ASHRAE) (2014).

[14] Piro M., Kämpf J.H., Ballarini I., Corrado V. (2026). A scalable, automatic, and evolutionary algorithm for calibrating urban building energy models. Sustain. Cities Soc..

[15] Granderson J., Touzani S., Custodio C., Sohn M.D., Jump D., Fernandes S. (2016). Accuracy of automated measurement and verification (M&V) techniques for energy savings in commercial buildings. Appl. Energy.

